# Effectiveness and Safety of Pharmacological Treatments for Chronic Inducible Urticaria: A Narrative Synthesis

**DOI:** 10.3390/jcm15197685

**Published:** 2026-10-04

**Authors:** Eleni Chatzidimitriou, Elisavet Tsiogka, Aikaterini Tsiogka, Ioannis-Alexios Koumprentziotis, Michael Makris, Dimitrios Rigopoulos, Alexandros I. Stratigos, Stamatios Gregoriou

**Affiliations:** 1 1st Department of Dermatology and Venereology, Medical School, National and Kapodistrian University of Athens, “A. Sygros” Hospital for Skin and Venereal Diseases, 16121 Athens, Greece; 2 Allergy Unit, 2nd Department of Dermatology and Venereology, National and Kapodistrian University of Athens, University General Hospital ‘Attikon’, 12462 Athens, Greece; 3 Hygeia Hospital, 15123 Athens, Greece

**Keywords:** physical urticaria, inducible urticaria, treatment, dermographism, cold urticaria, cholinergic urticaria, omalizumab

## Abstract

**Background**: Several treatments for chronic inducible urticaria (CIndU) have been studied, but reviews covering all subtypes are limited. **Objectives**: Our goal is to synthesize the effectiveness and safety of available therapies across all CIndU subtypes. **Methods**: We performed a review of PubMed and Scopus databases to identify studies reporting treatment data on cold urticaria, symptomatic dermographism, solar urticaria, delayed-pressure urticaria, exercise-induced urticaria, cholinergic urticaria and mixed subtypes. No studies were identified that specifically focused on heat, aquagenic, or contact urticaria; however, these subtypes are encompassed within certain studies examining mixed subtypes. **Results**: Our search yielded 36 studies. Second-generation H1-antihistamines as first-line treatment provided significant symptom control in most CIndU subtypes, with updosing to four times the standard dose showing greater effectiveness than standard doses. Omalizumab was highly effective as second-line treatment for antihistamine-refractory patients, although relapse after discontinuation was common. In delayed-pressure urticaria dapsone and high-dose intravenous immunoglobulin yielded good responses. Narrowband UVB benefited symptomatic dermographism and solar urticaria but with high relapse rates. Emerging mast cell-depleting therapies (barzolvolimab, lirentelimab) showed promising effectiveness, while the interleukin-1 inhibitor rilonacept failed to demonstrate clinical benefit. Across the 36 studies, adverse events were generally low for most treatments, with higher incidence reported with barzolvolimab. **Conclusions**: Updosed antihistamines are more effective than standard doses in controlling CIndU symptoms without a significant increase in adverse events. Omalizumab at 150–300 mg every 4 weeks is effective for antihistamine-refractory CIndU, though it is off-label. Relapse after treatment cessation is frequent, highlighting the need for personalized long-term strategies. Novel endotype-specific treatments require dedicated CIndU trials, as current evidence is largely extrapolated from chronic spontaneous urticaria.

## 1. Introduction

Chronic inducible urticaria (CIndU) is characterized by the recurrent appearance of itchy wheals, angioedema or both in response to specific, identifiable and reproducible triggers. CIndU subtypes include symptomatic dermographism (SDerm), which is the most common type, cold urticaria (Cold U), delayed-pressure urticaria (DPU), solar urticaria (Solar U), heat urticaria (Heat U), vibratory angioedema, cholinergic urticaria (CholU), which is the second most frequent type, contact urticaria and aquagenic urticaria. Importantly, two or more subtypes can coexist in the same patient [1]. The prevalence of CIndU is estimated to be around 0.5% [2]. CIndU is a hard-to-treat disease that profoundly impairs quality of life, by restricting work, leisure and social life [3]. The international treatment algorithm for chronic urticaria recommends second-generation H1 antihistamines as first-line treatment, with doses ranging from the standard dose up to four times that amount. For patients who do not respond, the monoclonal anti IgE-antibody omalizumab is recommended, though it is currently licensed only for chronic spontaneous urticaria (CSU). Although dupilumab and remibrutinib are recommended as a second-line treatment for chronic urticaria and despite the growing amount of evidence, specific recommendations for the treatment of CIndU are lacking in the International Guideline for the Definition, Classification, Diagnosis and Management of Urticaria. Subsequently, cyclosporine may be considered in combination with a second-generation H1 antihistamine [1]. The aim of this narrative synthesis is to provide a comprehensive overview of the available treatments for CIndU.

## 2. Materials and Methods

### 2.1. Search Strategy and Eligibility Criteria

The PubMed and Scopus electronic database were searched for randomized controlled trials (RCTs), retrospective and prospective cohort studies and case series of a minimum 5 patients assessing the treatment of CIndU, including Cold U, SDerm, DPU, Solar U, CholU, Heat urticaria, Aquagenic urticaria and Contact urticaria from inception to February 2026. Search terms included: “physical urticaria or inducible urticaria or Cold U or SDerm or DPU or Solar U or CholU or Heat urticaria or Aquagenic urticaria or Contact urticaria AND treatment”. We included studies in real-world settings, since pivotal clinical trial data are limited. We included only studies in English language. Reference list from the included studies were also screened to identify any other relevant studies not yielded during the initial search. Full-text screening was conducted independently by two reviewers and disagreements were reassessed until agreement was reached. Consequently, the effectiveness rather than the efficacy of treatment interventions was evaluated. Studies reporting treatment outcomes in patients with both inducible and spontaneous urticaria were included only if clear outcomes of inducible urticaria alone were provided. All research was conducted according to a protocol registered in INPLASY (ID number: INPLASY202670084, DOI: 10.37766/inplasy2026.7.0084).

### 2.2. Data Extraction

Data were extracted by first author, year of publication, type of study, number of participants, type of treatment, primary endpoints and last follow-up period.

### 2.3. Study Selection

**Figure 1:**
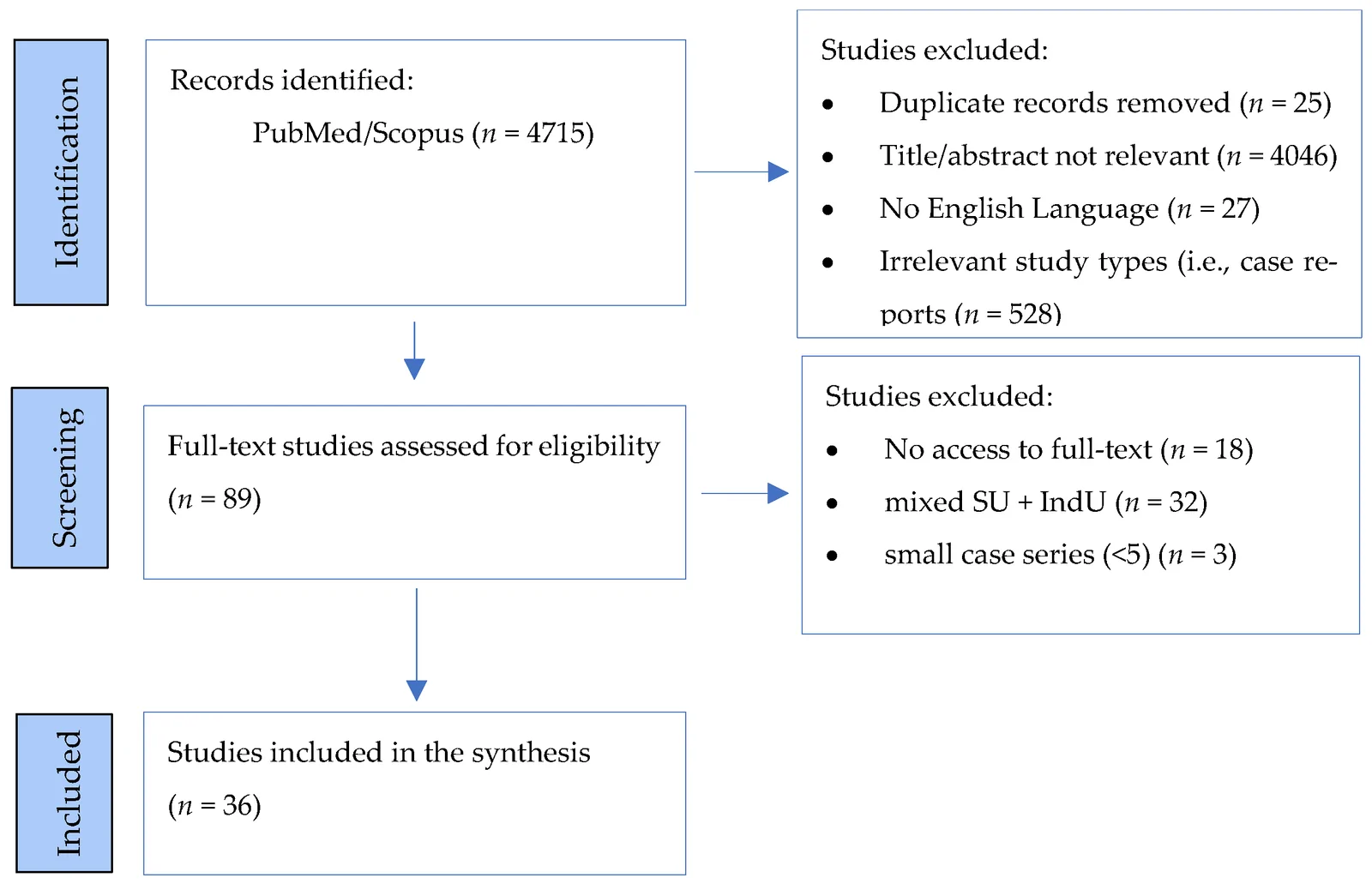
Flow diagram.

The initial search yielded 4715 articles (no filters used). Title and abstract identified 89 articles, which were selected for full-text review. Finally, 36 studies were included in the narrative synthesis after exclusion of irrelevant studies (Figure 1).

## 3. Results

Our synthesis included a total of 36 studies, comprising 8 on Cold U, 3 on SDerm, 3 on DPU, 4 on Solar U, 7 on CholU and 11 on CIndU with various combinations of subtypes. The results of all these studies are summarized in Table 1.

In Cold U four studies investigated second-generation antihistamines (levocetirizine, mizolastine, desloratadine and rupatadine; one study each), with cyclosporine added to levocetirizine in a subset of patients [4,5,6,7]. One study used doxantrazole (a mast cell stabilizer), one used rilonacept (Interleukin-1 inhibitor) and two evaluated omalizumab [8,9,10,11]. For diagnostic evaluation in those studies, six studies employed the ice cube provocation test, one used cold water immersion and two did not specify the method. Outcome measures included the Critical Stimulation Time Test (CSTT; minimum time to induce a wheal) in two studies, the Critical Temperature Threshold (CTT; highest reactive temperature) in two studies and the Dermatology Life Quality Index (DLQI) in one study. Overall, second-generation antihistamines enabled most patients to achieve an increase in CSTT and reduction in CTT. Omalizumab also demonstrated clinical improvement in 92.3% of children and 55.6% of adults with Cold U, based on subjective clinical assessment by the treating investigators [10,11]. Overall, the drugs were well tolerated; the most common adverse events were mild and transient (headache, fatigue, somnolence and injection-site reactions), with no serious events or treatment discontinuations.

Three studies on SDerm assessed omalizumab, psoralen and ultraviolet A (PUVA) and narrowband UVB [12,13,14]. The first study used the FricTest and the DLQI; the other two used a calibrated dermographometer, with one also employing a Visual Analog Scale (VAS) to assess lesion number and size. In the omalizumab study, approximately half of the patients achieved a complete response (grade 4 on FricTest), while the other two interventions showed outcomes ranging from no effect using PUVA to substantial symptom reduction (itch and wheal) using NB-UVB. Omalizumab caused upper respiratory infections and headache, whereas PUVA and narrowband—ultraviolet B (NB-UVB) only produced mild erythema; no serious events or withdrawals occurred in any group.

In DPU the first study investigated the addition of theophylline to cetirizine, with outcomes assessed using the VAS, an 11-point treatment effectiveness score (TES) and pain scales [15]. The second evaluated dapsone combined with vitamin C, employing the 11-point Numeric Rating Scale (NRS-11) to measure symptom improvement [16]. The third examined high-dose intravenous immunoglobulin (IVIG); diagnosis was based on an unequivocal clinical history and typical lesions without a provocation test and the autologous serum skin test (ASST) was used to screen for histamine-releasing autoantibodies [17]. Overall, the symptoms improved considerably as assessed by the aforementioned patient-reported outcomes. Theophylline had no reported adverse effects; dapsone caused dose-related hematological issues (hemolysis, methemoglobinemia), potentially affecting outcomes or causing discontinuation; IVIG induced transient mild symptoms (headache, fatigue, fever, nausea) resolving within 24 h, with no serious events or withdrawals.

Ιn solar urticaria three studies assessed the effects of afamelanotide (a melanin-stimulating α-MSH analog), IVIG and NB-UVB phototherapy [18,19,20]. Outcome evaluation relied on phototesting in all three, while two of them further determined the minimum urticarial dose (MUD)—the lowest ultraviolet dose that induces a visible wheal. The fourth study evaluated omalizumab, with outcomes assessed using the Urticaria Control Test (UCT) [21]. The first three studies demonstrated either an increase in MUD or improved sun tolerance, whereas the omalizumab study reported reaching 90%. Omalizumab had no reported adverse events and no treatment discontinuations; in contrast, afamelanotide caused only a mild unrelated upper respiratory tract infection, IVIG led to aseptic meningitis in one patient and NB-UVB produced mild erythema, occasional solar urticaria relapses and painful marked erythema in a minority—yet none of these treatments raised serious safety concerns or led to withdrawals.

In CholU three studies investigated second-generation antihistamines (mainly cetirizine), two of which used increased doses. Among these, two assessed outcomes using daily diary cards (daily symptom scores recorded by patients), while the third utilized the CholUAS7 score, the DLQI and a provocation test. In all three studies, a reduction in disease activity was observed, with improvement in pruritus, wheals and erythema [22,23,24]. Four studies evaluated omalizumab. In two of these, study-specific indices (e.g., UCOL score) were described; three employed the VAS; two used the DLQI; and one used the urticaria activity score 7 (UAS7). Overall disease improvement was observed as assessed by the utilized disease assessment scores and provocation tests [25,26,27,28]. Cetirizine was well tolerated, causing mild transient side effects (diarrhea, anorexia, tiredness) in a few patients and none with high-dose use, whereas omalizumab had mild suspected events (headache, myalgia, fatigue, injection reactions) with no severe adverse events or withdrawals.

Eleven studies investigating patients with CIndU across various subtype combinations were identified. The number of studies for each subtype was as follows: SDerm, 11; CholU, 8; Cold U, 10; heat urticaria, 4; DPU, 5; aquagenic, 3; Solar U, 3; contact urticaria, 1. Two studies used second-generation antihistamines (cetirizine, ebastine) [29,30]. The rest investigated monoclonal antibodies (lirentelimab, 1 study; omalizumab, 7 studies; barzolvolimab, 1 study) [31,32,33,34,35,36,37,38,39]. Regarding the study results, the UCT index was the most used tool (7 studies), followed by the UAS7 (2 studies) and the DLQI (2 studies). Depending on the subtype of urticaria, the corresponding provocation tests described earlier were selected. Overall, second-generation antihistamines improved patients’ symptoms, whereas in the studies with monoclonal antibodies, a complete response rate ranging from 41% to 100% was observed according to the UCT. Cetirizine and ebastine were well tolerated, with only mild transient effects (tiredness, headache, dry mouth, somnolence, fatigue) and no serious adverse events reported. Lirentelimab was associated with transient infusion reactions, nasopharyngitis and headache; omalizumab was well tolerated, with only mild side effects and few discontinuations; and barzolvolimab proved similarly favorable, causing only mild transient events, reversible hair and taste changes and asymptomatic blood count decreases that required no intervention—critically, no serious adverse events or dose-limiting toxicities were reported across the three agents.

**Table 1:** Included studies.

1st Author, Year	Study Type	*n*	Treatment Type	Results	Last Follow-Up
Cold Urticaria (Cold U)					
Mehta et al., 2025 [4]	Retrospective cohort	15	Levocetirizine+ Cyclosporine	Ice cube provocation test: 40% achieved complete control with standard-dose second-generation antihistamines. 40% required up-titration of antihistamines (2× to 4× standard dose) to achieve remission. 20% were refractory to high-dose antihistamines and received cyclosporine, all achieving remission within 4–10 weeks.	47 months
Bentley-Phillips et al., 1978 [8]	Double blind non-randomized cohort	13	Doxantrazole	Primary cold provocation was cold water immersion: reduce cold-induced histamine release but the effect was often incomplete, leading researchers to suggest it might be best used in combination with other therapies.	ND *
Metz et al., 2010 [7]	RCT	21	Rupatadine	Significantly increased the CSTT. Complete response: 52% of patients showed no wheal after a 5 min ice cube test. CTT significantly reduced by a median of 8 °C whereas placebo had no effect. Wheal scores significantly reduced. Pruritus was absent in 67%.	42 days
Dubertret et al., 2003 [5]	RCT	28	Mizolastine	CSTT: significantly increased. Mizolastine significantly reduced the mean wheal size at 3 min and 10 min after ice cube challenge. Pruritus intensity was significantly reduced. A significantly higher proportion of patients experienced at least a 50% reduction in total papular response scores with mizolastine.	28 days
Fialek et al., 2021 [10]	Retrospective cohort	14 (only ColdU)	Omalizumab	300 mg every 4 weeks achieved complete response in 55.6% of patients at 3–6 months. All patients who stopped treatment eventually relapsed but retreatment was effective.	ND *
Bonnekoh et al., 2023 [9]	RCT	20	Rilonacept	CTT: The difference was not statistically significant. DLQI showed a significant improvement in the rilonacept group after 6 weeks. Long-term treatment with rilonacept (up to 12 weeks) does not improv cold urticaria disease activity.	16 weeks
Briand et al., 2021 [11]	Retrospective case series	13	Omalizumab	12 out of 13 children (92.3%) showed clinical improvement based on subjective clinical assessment by the treating investigators. Time to response variable, most of them in one month. Relapse is common after discontinuation, but retreatment had good effectiveness.	67 months
Juhlin, 2004 [6]	Prospective cohort	12	Desloratadine	No wheals occurred in any patient after 12 min of ice application. Only 2 patients had ≥4 small wheals after 12 min. Mean time increased to 7.0 min (after) in 7 patients. No itching occurred in any patient. All patients reported better cold tolerance in daily life. Dose escalation in partial responders needed.	3 months
Symptomatic Dermografism (SDerm)					
Mauer et al., 2017 [12]	RCT	61	Omalizumab	Based on FricTest: Both omalizumab doses (150 mg/300 mg) significantly improved friction thresholds from week 4. Complete response rates (week 10): Placebo: 11% Omalizumab 150 mg: 44% Omalizumab 300 mg: 53% Clinically meaningful improvement (≥4-point DLQI reduction) Response is rapid (as early as 4 weeks) but not sustained after discontinuation (relapse within 6 weeks).	16 weeks
Logan et al., 1989 [13]	Prospective cohort	14	PUVA	Based on a calibrated dermographometer: No significant reduction in itching, in wheal width was observed. No consistent difference in erythema (flare) area.	3 months
Borzova et al., 2008 [14]	Prospective cohort	8	Narrowband ultraviolet B phototherapy (NB-UVB)	Based on a calibrated dermographometer: Mean reduction in VAS itch score was 52.3%. Mean reduction in VAS wheal score was 71%. Itch and wheal scores showed significant relapse at 12 and 18 weeks post-treatment, but objective wheal measurements did not.	18 weeks
Delayed-Pressure Urticaria (DPU)					
Kalogeromitros et al., 2005 [15]	Prospective cohort	23	Addition of theophylline to cetirizine treatment	1 month: Only burning/pain improved significantly. 2 months: Burning/pain remained improved. 3–4 months: All variables (VAS, TES, pain) improved significantly. 5 months: Improvement persisted but began to decline slightly.	12 months
Grundmann et al., 2011 [16]	Retrospective cohort	15	Dapsone with vitamin C	Using NRS-11 for symptom improvement response rate: 93% achieved a “good” or “very good” treatment response 27% had complete symptom resolution. The highest response rate (90%) was seen with 8–12 weeks of dapsone therapy.	Mean 9.7 ± 5.2 weeks (range not specified)
Dawn et al., 2003 [17]	Retrospective case series	8	IVIG	Overall response: 3 patients achieved remission; 2 patients improved; 3 patients unchanged. ASST results: 3 patients were ASST-positive; 2 patients had delayed positive ASST; 3 were ASST-negative. Benefit was often seen after 1–3 infusions; additional infusions beyond three offered limited advantages.	Range 12–48 months
Solar Urticaria (Solar U)					
Haylett et al., 2011 [18]	Phase II, open-label non-randomized study	5	Afamelanotide (synthetic α-MSH analog that stimulates melanin production)	Based on monochromated light testing: Significant melanin rise by day 7, peaking at day 15, sustained at day 60. Increased in both exposed and unexposed skin. MUD increased in all patients at 30 and 60 days. Weal area and flare area reduced at 30 days. Broad-spectrum protection observed (UVA, UVB, visible light).	60 days
Adamski et al., 2011 [19]	Retrospective cohort	7	Intravenous Immunoglobulins (IVIG)	Based on polychromatic phototesting and UVA phototesting: UVA MUD increased in 5 out of 7 patients. IVIG produced 71% complete remission rate. Remission was maintained for 4 to more than 12 months, although antihistamines were still occasionally needed.	12 months
Casanova-Esquembre et al., 2024 [21]	Retrospective cohort	13	Omalizumab	Complete clinical response (UCT = 16): 9 out of 10 patients with available follow-up data (90%).	Mean of 35 months
Calzavara-Pinton et al., 2012 [20]	Prospective cohort	39	Group A: Received standard regimen NB-UVB Group B: Received an intensive regimen NB-UVB	Based on phototesting with broadband UVB, narrowband UVB, UVA and visible light: Most patients reported improved sun tolerance after treatment. The intensive regimen provided more durable protection (stable at 3 months) compared to standard regimen (progressive loss). At 1-month follow-up: 13.8% of Group A had relapses; 20% of Group B had relapses. At 3-month follow-up: 20.7% of Group A had relapses; no new relapses in Group B.	3 months
Cholinergic Urticaria (CholU)					
Koch et al., 2016 [22]	Prospective cohort	28	Second-generation antihistamine (sgAH) at 4× standard dose	Significantly reduce disease activity but only 39% achieved ≥50% improvement in their CholUAS7 score. Itch responds better than wheals. DLQI: 21.6% improvement. PCE provocation test (UASprovo): 12.8% improvement.	No long-term follow-up after treatment cessation
Zuberbier et al., 1995 [23]	RCT	23	Cetirizine	Patient daily diary cards (daily symptom scores): Highly significant results for pruritus and whealing. PCE test proved to be impractical for monitoring treatment effectiveness due to poor reproducibility.	1 week
Altrichter et al., 2019 [25]	Retrospective cohort	11 - isolated CholU - CholU + ColdU	Omalizumab	Response was measured using a qualitative scale based on the physician’s clinical evaluation, not a standardized scoring tool. CholU + ColdU: 1 patient Complete or Major Response for both 1 patient Pure + No Response. Isolated CholU: 5 patients Complete or Major Response; 4 patients Pure + No Response; Responses maintained for up to 26 months with continued treatment.	26 months
Ghazanfar et al., 2020 [26]	Prospective cohort	18 - isolated CholU - CholU + other CIndU	Omalizumab	Isolated CholU showed reduction in disease activity (UAS7 improvement) and significant improvements in both DLQI and VAS scores. Patients with CholU and other CIndU demonstrated numerical improvements across all outcomes.	6 months
Zuberbier et al., 1996 [24]	RCT	13	Cetirizine high dose	Using daily diary card scoring demonstrated statistically significant improvement across all symptoms (Wheals, Erythema, Pruritus showed the most substantial improvement) compared to placebo.	3 weeks
Kim et al., 2020 [27]	Retrospective cohort	21 isolated cholU	Omalizumab	Using VAS: Complete response: 4.8%. Partial response: 57.1%. Most responders improved within 4 weeks, though some required longer treatment (≥3 months).	ND *
Gastaminza et al., 2019 [28]	RCT	22	Omalizumab	Primary outcome: Overall negative challenge rate at week 48: 31.3%. UCOL Score, Quality of Life (CU2-QoL), VAS Score and Days with Symptoms (per month): significant improvement after 8 doses (week 48). Slow onset of action starting from the 4th dose (16 weeks). Relapse after discontinuation. All outcome measures worsened significantly within 3 months of stopping treatment.	60 weeks
Inducible Urticaria with variant combinations of subtypes					
Juhlin et al., 1987 [29]	Prospective cohort	22 - SDerm - ColdU	Cetirizine	All assessments were performed at a single time point: 4 h after medication intake. Complete inhibition possible: 8/10 dermographism patients (by using Dermographometer) and 5/12 cold urticaria patients had complete wheal suppression (ice cube applied). All patients with itching experienced complete relief. The redness/flare response was not inhibited.	No longer-term follow-up was conducted in this study
Qian et al., 2022 [30]	Prospective	103 - SDerm - CholU - ColdU	Ebastine	Authors created their own urticaria symptom severity score: 99% of inducible urticaria patients achieved marked effect with ebastine (80.6% at 10 mg, 12.6% at 20 mg, 5.8% at 40 mg).	1 month
Altrichter et al., 2022 [31]	Phase IIa, open-label, study	21 - CholU - SDerm	Lirentelimab	CholU: 82% complete response (UCT); 100% had negative exercise challenge test (PCT); 36% achieved CholUAS7 = 0. SDerm: 40% complete response (UCT); 60% complete itch resolution (FricTest); 40% complete wheal resolution (FricTest); Improvements were sustained through the 22-week treatment period, with a rebound after treatment cessation.	28 weeks
Soegiharto et al., 2025 [32]	Retrospective cohort	178 - CholU - Cold - SDerm - DPU - Solar - Heat - Aquagenic - Contact	Omalizumab	41% complete control and 37% well-controlled based in UCT; 26% discontinued omalizumab.	Maximum observation period was 13 years
Buters et al., 2021 [33]	Retrospective cohort	44 - DPU - CholU - SDerm - Cold - Heat - Aquagenic	Omalizumab as add-on to antihistamines	Standard omalizumab dosing: UCT 50% complete or good control 12 weeks. A personalized approach (updosing or interval extension) increased the proportion with complete or good control to 66%. Relapse after discontinuation was common.	1 year
Yu et al., 2021 [34]	Retrospective cohort	59 - SDemr - CholU - Cold	Omalizumab	UCT: 78.0% well-controlled disease. UCT: 55.9% complete response, among them 60.6% within the first 2 months. 59.3% achieved DLQI of 0 or 1 (no QoL impairment). Relapse after discontinuation was common but retreatment was effective after the first administration.	6 months
Terhorst-Molawi et al., 2023 [39]	Phase 1b, open-label study	21 - Cold - SDemr	Barzolvolimab	ColdU: 100% complete response-negative provocation test (TempTest). SDerm: 90% complete response-negative pins (FricTest). 100% achieved well-controlled disease (UCT ≥12). 90% achieved DLQI scores of 0–1.	36 weeks
Metz et al., 2011 [35]	Case series	7 - Solar - SDerm - Cold - DPU - Heat	Omalizumab	5 of 7 patients achieved complete symptom control within days after the first injection confirmed by negative provocation tests. Relapse occurred after treatment interruption, but retreatment was again rapidly effective.	Variable, patient-specific follow-up
Litovsky et al., 2023 [36]	Retrospective cohort	89 - CholU - SDerm - Cold	Omalizumab	UCT+UAS7: 56.3% complete response; 14.9% partial response; 18.4% no response. No response led to earlier treatment stop.	Median follow-up of 2.3 years
Jeong et al., 2022 [37]	Retrospective cohort	29 - CholU - SDerm - Cold - DPU	Omalizumab	Overall responder rate: 62.1% (based on UAS7). Significantly improved within 4 weeks, with continued improvement through 12 weeks.	12 weeks
Exposito-Serrano et al., 2021 [38]	Retrospective cohort	80 - Solar - Cold - SDerm - CholU - DPU - Aquagenic - Heat	Omalizumab	UCT: 72.5% complete or well response, among them 57% <3 months. Updosing rescued about half of initial non-responders. Relapse occurred after treatment interruption, but retreatment was again rapidly effective.	ND *

* ND: Not documented.

## 4. Discussion

Our narrative synthesis included 36 studies (1118 patients) on Cold U, SDerm, Solar U, DPU, CholU and CIndU with variant subtype combinations. No studies were identified on other forms of CIndU (heat, contact, aquagenic urticaria or vibratory angioedema). Despite the plethora of individual studies investigating specific subtypes of CIndU, the present synthesis summarizes available treatment data on all subtypes of CIndU. In contrast to the many RCTs and real-life data on CSU that provide a huge body of evidence for many different therapeutic interventions, data on CIndU are still lacking and this is reflected in the recent International Guidelines, which extrapolate data from CSU in their recommendations for the pharmacological treatment of CIndU. The present narrative synthesis summarizes available treatment data on all subtypes of CIndU.

The mainstay of managing CIndU involves defining relevant triggers via provocation threshold testing to facilitate avoidance, a step that is particularly critical for subtypes with systemic symptoms or anaphylaxis, although it is not always fully achievable. As emphasized by McSweeney et al. and Maltseva et al., provocation tests are essential not only for diagnosis but also for the quantitative assessment of disease severity and objective monitoring of treatment response. They also serve as the gold standard in clinical trials, offering reproducible endpoints for reliable treatment comparisons and personalized care, while compensating for the limited availability of validated disease-specific Patient-Reported Outcome Measures [40,41].

Second-generation antihistamines remain the first-line treatment; updosing to four times the standard dose provides significant symptom control with only mild and transient adverse events reported in most CIndU subtypes. Personalized dosing strategies and the use of serum tryptase as a biomarker are also supported [4,5,6,7,15,23,24,29,30]. However, in the study by Koch et al., this approach yielded only 39% of the patients reported an improvement in CholUAS7 disease activity score of 50% or greater [22].

A total of 16 studies investigated omalizumab across multiple CIndU subtypes. The findings demonstrate that omalizumab is highly effective and safe as a second-line treatment for patients unresponsive to high-dose antihistamines. In Cold U, omalizumab 300 mg every 4 weeks achieved clinical improvement raging from 55.6 to 92.3%, although recurrence upon discontinuation is common [10,11]. For SDerm both 150 mg and 300 mg doses rapidly improved friction thresholds and quality of life, but benefits were transient after treatment discontinuation [12]. In Solar U, omalizumab produced a 90% complete clinical response rate [21]. For CholU, omalizumab was safe and effective, yet the response was slow and progressive, often requiring over four months to fully evaluate; after prolonged administration, about 31.3% of patients achieved a negative exercise challenge test, with female patients responding more favorably than males [25,26,27,28]. Overall, omalizumab seems to be highly effective and safe across both chronic spontaneous and inducible urticaria, but relapse is frequent after cessation, making retreatment effective and personalized long-term strategies essential.

The combination of ciclosporin with a modern second-generation H1 antihistamine is off-label for urticaria and is recommended only for patients with severe disease that is refractory to all other licensed treatments. Mehta et al. demonstrated that ciclosporin, as an add-on therapy to maximum-dose antihistamines, is a viable treatment option for refractory ColdU. This subgroup demonstrated a favorable and rapid response, with clinical improvement observed within four weeks and complete remission achieved by ten weeks of treatment [4]. However, due to a higher incidence of adverse effects—particularly with long-term use—this drug cannot be recommended as standard treatment.

In SDerm NB-UVB phototherapy reduced itching and whealing but is associated with a high relapse rate within three months, while PUVA is not justified due to lack of effectiveness [13,14]. In DPU dapsone was highly effective (93% good or very good outcomes) with sustained benefits even after discontinuation; high-dose IVIG induced remission with three or fewer infusions and theophylline served as an add-on therapy in cetirizine treatment, requiring at least two months to become statistically significant [15,16,17]. In Solar U, NB-UVB has been shown to act as a prophylactic measure, IVIG provided a 71% remission rate and afamelanotide offered systemic photoprotection [18,19,20].

Bonnekoh et al., studied rilonacept (Interleukin-1 inhibitor) in cold urticaria. Rilonacept failed to significantly reduce CTT in patients although it produced a temporary, non-sustained improvement in DLQI [9].

In the study by Altrichter et al. (2022), lirentelimab (which selectively inhibits mast cells and depletes eosinophils) demonstrated clinical activity across antihistamine-refractory chronic urticaria subtypes, including omalizumab refractory CSU, CholU and SDerm [31]. Terhorst-Molawi et al., showed that barzolvolimab demonstrated rapid, durable mast cell depletion, even in patients with CIndU refractory to omalizumab. For Cold U barzolvolimab achieved a 100% complete response rate (10/10 patients) and for SDerm the complete response rate was 90% (9/10 patients) [39].

Several emerging therapies targeting endotype-specific pathways—including anti IgE, Bruton’s tyrosine kinase inhibitors, dupilumab (anti IL 4Rα), other anti-cytokine drugs, Janus Kinase inhibitors and KIT mediated mast cell depletion (e.g., barzolvolimab)—are currently in clinical development and may offer superior effectiveness to existing treatments. However, nearly all trials involving these novel agents focus on CSU [1]. This highlights the pressing need for further research on CIndU.

## 5. Limitations

Several limitations should be considered when interpreting these findings. Sample size calculations were not reported in several studies and some included relatively small cohorts. Consequently, these studies may have been underpowered to detect clinically differences between groups and statistically nonsignificant findings should not necessarily be interpreted as evidence of no effect. Second, substantial heterogeneity was observed across the included studies, particularly with respect to the assessment methods, outcome measures, endpoints and timing of evaluations. Differences in study design, participant characteristics, interventions and follow-up periods may have further contributed to variability in the reported findings. This methodological and clinical heterogeneity limited the comparability of results across studies. Similarly, where outcomes were assessed using different scales or at different time points, apparent differences between studies may reflect methodological variation rather than true differences in treatment effects. These limitations reduce the precision and generalizability of the findings. Future studies would benefit from adequately powered prospective designs, standardized outcome definitions and assessment methods, consistent follow-up intervals and predefined clinically relevant endpoints.

## 6. Conclusions

CIndU remains insufficiently researched, highlighting the need for treatments that prevent, delay, or slow the progression of symptoms and induce disease remission. Nevertheless, the findings support the potential effectiveness of the stepwise treatment algorithm recommended by international guidelines for CIndU [1]. This approach involves second-generation H1-antihistamines as first-line treatment, with dose escalation up to four times the standard dose. For patients who do not respond to antihistamines, omalizumab—proposed as second-line treatment—appears to be an effective option, although its use in CIndU remains off-label. Further studies are required to assess the effectiveness and safety of novel and investigational treatments of CSU also in CIndU.

## Data Availability

The authors confirm that the data supporting the findings of this study are available within the article.

## Abbreviations

The following abbreviations are used in this manuscript:

ASST	autologous serum skin test
CholU	cholinergic urticaria
CIndU	chronic inducible urticaria
Cold U	cold urticaria
CSTT	Critical Stimulation Time Test
CSU	chronic spontaneous urticaria
CTT	Critical Temperature Threshold
DLQI	Dermatology Life Quality Index
DPU	delayed-pressure urticaria
Heat U	heat urticaria
IVIG	intravenous immunoglobulin
MUD	minimum urticarial dose
NB-UVB	narrowband—ultraviolet B
NRS	Numeric Rating Scale
PUVA	psoralen and ultraviolet A
RCT	randomized controlled trial
SDerm	symptomatic dermographism
Solar U	solar urticaria
TES	treatment effectiveness score
UAS7	urticaria activity score 7
UCT	Urticaria Control Test
VAS	Visual Analog Scale
